# Self-reported drinking and driving amongst educated adults in Spain: The "Seguimiento Universidad de Navarra" (SUN) cohort findings

**DOI:** 10.1186/1471-2458-7-55

**Published:** 2007-04-12

**Authors:** Maria Segui-Gomez, Silvia Palma, Francisco Guillen-Grima, Jokin de Irala, Miguel A Martinez-Gonzalez

**Affiliations:** 1 Department of Preventive Medicine and Public Health, Universidad de Navarra, Pamplona, Spain; 2 Department of Health Sciences, Universidad de Jaén, Jaén, Spain; 3 Preventive Medicine Unit, Clínica Universitaria, Universidad de Navarra, Pamplona, Spain

## Abstract

**Background:**

The role of alcohol as a risk factor for motor vehicle crashes is long known. Yet, reports on the prevalence of drinking and driving suggest values between 20%–30% when the adult driving population is interviewed. We wondered whether these values hold true among European educated citizens and whether there are any significant differences in prevalence by age, gender, type of profession and other lifestyle indicators.

**Methods:**

Cross-sectional analyses of baseline data from a cohort of university graduates in Spain (SUN study). Answered questionnaires contained items on current drinking and driving practices, together with data on socio-demographic characteristics and lifestyle habits. Chi square, Fisher test, and multivariate logistic regression were used to investigate the impact of several variables on drinking and driving practices. Analyses were stratified by gender.

**Results:**

Almost 30% of the participants reported "sometimes" drinking and driving. This percent increased to 47% when "almost never" was also included as a positive answer to the drinking and driving practice question. These percentages varied significantly by gender, with up to 64% of men reporting "sometimes" or "almost never" vs. 36% of women doing so. Drinking and driving practices also differed by overall alcohol consumption habits, smoking, use of safety belts, and notably, type of profession.

**Conclusion:**

Our findings are amongst the first on the high prevalence of drinking and driving among Spanish. Particularly worrisome is the fact that health professionals reported this habit even at higher rates. Multidisciplinary interventions (e.g., legal, educational, economic) are needed to reduce this serious health risk.

## Background

Motor vehicle crashes constitute one of the leading mechanisms of death and disability around the world. The WHO estimates that 1.2 million people die on a yearly basis in relation to these crashes. They further estimate that between 20 and 50 million people are injured or disabled because of non-fatal motor vehicle injuries [1]. In Europe, this means 127,000 annual deaths, 2.4 million injured people and costs exceeding 2% of the European Gross Domestic Product [2].

The role of alcohol in this public health problem has been known for quite some time; alcohol increases both the chances of being involved in a crash and the severity of injuries sustained once a crash occurs [3,4]. Drivers with Blood Alcohol levels as little as 0.02–0.05 g/100 ml sustain three times more chances of being killed in a single vehicle crash than drivers with no alcohol at all; an increase that goes up to 52 when alcohol levels are 0.08–0.1 in 16–20 years old males, an amount easily reached with three cans of beer if the subject weights 70 kg [5]. In the US, it is estimated that 41% of drivers killed in crashes were involved in alcohol-related crashes; a percentage similar to the 37% of occupants killed in this type of crashes. In addition, 44% of motorcyclist and 47% of pedestrian deaths are associated with alcohol-related crashes [4]. In New Zealand, 30% of crash injuries are attributable to alcohol [3]. Studies conducted in European countries other than Spain quantify the number of crash fatalities related to alcohol in a range from a high 40% in Ireland [6] to a low 14% in the United Kingdom [7] or Germany [8]. In Spain, it is estimated that between 30% and 50% of road traffic deaths are due to alcohol-related crashes [9].

Recent trend analyses suggest that there has been a halt in the progress to reduce this problem in recent years [4,10,11]. Among the available studies, mostly from representative samples of the US adult population, findings range from the 31% of US drivers aged 16 or older who self-report driving within two hours after drinking [12] to the 25.7% of respondents who report driving after having had one or more drinks during the month prior to the interview [13,14], or to the 24% of men and 9% of women doing so when asked a similar question [15]. The Royal 2000 study calculates that 82 million drinking-driving trips are done in the US in a given year with blood concentration levels at the legal limit or higher, which amounts to 10% of all drinking and driving trips. Yet, Dellinger et al (1999) [16] and Quinlan et al (2005) [11], using different datasets that are drawing representative samples of the adult US population, quantify in 159 million the number of annual alcohol-impaired trips from 1999 to 2002. In particular, the Dellinger estimate is derived after identifying that 3% of respondents declare drinking and driving within the 30 days prior to the survey (4.8% among male, 1.3% among female subjects), while 4.9% of passengers report having ridden with a driver who had drunk [16]. A survey of university students from New Zealand quantifies in 8.4% of male and 3.4% in female students who report to have drunk and driven during four weeks prior to the interview and an 11.5% and 7.0% of women who report drink-riding [17]. However, another New Zealand finding, reported from the Blood Donor's Health study, suggests that 21% of the participants had driven a motor vehicle when they considered themselves over the legal limit for alcohol [18].

European data from a pool of interviewees from 13 countries in 2000 in relation to the European and International Health and Behaviour Surveys suggest that 21% of safety-belt non users report to drive alcohol-impaired at least once in a year prior to the interview, whereas this percent was 11% among safety-belt users [19]. A comparison of police-based data on drinking and driving from seven countries (not including Spain) and for several years suggest that at least in four of them (US, Great Britain, Canada and the Netherlands) drinking and driving practices are stagnated or even increased in recent years [8]. Unpublished findings from another 2002 European survey of 23 countries, SARTRE-3, suggest that 43% of Spanish drivers report to have combined alcohol and driving within the week prior to the interview (Marie B. Biecheler-Fretel, personal communication). Unfortunately, producing aggregated European estimates of this habit with either these data or the one in the paper by Sweedler and colleagues [8] is not recommended given the methodological differences in data collection procedures across European countries.

Thus, we set ourselves to investigate the self-reported prevalence and trends of drinking and driving and whether there are differences in this behaviour by gender, profession and other lifestyle variables. We also hypothesised that, recent debates on the epidemic of motor vehicle crashes may have influenced on the prevalence rates, especially among better educated segments of the Spanish population.

## Methods

The "Seguimiento Universidad de Navarra" (SUN) study is a multipurpose cohort started in late 1999. Details on the cohort design, recruitment strategy and follow-up methods are available elsewhere [20]. Briefly, university graduates from Spanish universities are invited to participate in the study. The first invitees were graduates from the University of Navarra and members of the Alumni Association, an organization grouping more than 20,000 former students currently living throughout Spain. Soon other Spanish universities' graduate organizations agreed to invite their alumni (e.g., Universities of Cantabria, Zaragoza, and Jaen). Another strategy to enroll participants is through professional associations. In Spain all professionals (whether practicing in private or public settings) are mandated to register in their provincial association of trade (e.g., the Madrid "province" association of veterinarian doctors, or the Barcelona "province" association of nurses). We have invited members of several such professional regional associations. Because every year there are new graduates, and because the number of professional regional organizations is large (Spain is comprised of 49 "provinces" and there are many times the many trades – nurses, dentist, veterinarians, lawyers, architects, mechanical engineers....), we maintain an open enrolment style to accommodate both economically and logistically the ongoing recruitment efforts. As with any cohort study, our goal is not representativeness but fidelity. The participants in the cohort share the commonality of being university graduates and working or residing in Spain. As of 2006, the retention rate of the participants who have remained such for at least 4 years is 86% [21].

Once graduates agree to participate voluntarily and without any compensation, they complete the baseline questionnaire and are followed through biannual questionnaires which include an array of questions related to demographics, health state, lifestyle habits, and changes of these characteristics/variables over the time. Among the health endpoints under consideration, we include motor vehicle crashes involving hospital admission for at least 24 hrs.

Of interest to our hypotheses, the first (or baseline) questionnaire included several questions related to alcohol consumption. Specifically, there were two questions regarding how many days in a week alcohol was consumed (including and excluding consumption during mealtime), three questions regarding maximum number of drinks on a time (whether during the week, on a weekend, or on a special occasion), and five items of a Food Frequency Questionnaire devoted to the amounts of an assortment of alcoholic beverages drunk. Combining their answers to these questions, we defined occasional binge drinkers as those having had more than five drinks in a session (including weekends and special events). Last, there was a question regarding whether the subject "drive [s] when he/she has had any alcohol". Answers to this question could be "I do not drive", "absolutely never", "yes, sometimes", and "almost never".

Other questions of relevance were related to average kilometres driven per year, use of safety belt, and lifestyle indicators such as weight and height (the self-reports have been validated against objective measurements) [22], smoking status and others.

For our analysis we used all baseline questionnaires of participants who had joined the cohort prior to February 2006.

Since alcohol consumption, exposure to motor vehicles and involvement in motor vehicle crashes are so closely related to gender, we conducted stratified analyses by gender. Fisher 2-tail tests, Pearson chi-square tests, and chi square tests for trend were used for the comparisons of proportions between groups. Multivariate logistic regression models were created to evaluate the role of several socio-demographic and lifestyle characteristics in the probability of reporting drinking and driving.

Last, since enrolment in the cohort is open, we investigated whether subjects enrolling in different years reported differences in the practice of drinking and driving.

Statistical significance was defined at a p < 0.05. SPSS version 12.0.1 was used for analyses (SPSS Inc. 233 S. Wacker Drive, Chicago, IL)

## Results

Data were available from 16,171 university graduates who had enrolled in the cohort at the time of this analysis and their data had been cleaned and processed, 59.4% of them were women (N = 9,613). The average age of participants was 38 years old (SD 12.4). The vast majority of participants were drivers (92.4%), although this percent differed significantly by gender, with a larger proportion of men being drivers (96.6% vs 89.5%, p < 0.05). The average alcohol intake was 6.9 g/d (SD 11.2), although men had a higher average alcohol intake than women (mean = 11, SD = 15 vs. mean = 4 and SD = 6). Almost a third of the participants reported on occasional binge drinking, although men reported higher prevalence of such practice (39.8% vs. 22.0%, p < 0.001). Although 5% of participants reported ever requiring hospital admission in relation to a motor vehicle crash, this prevalence differed also by gender, being 6.6% among men and 4.3 among women (p < 0.001). Slightly more than half of the participants (52.8% or 8531) reported to never drink and drive. This differed by gender, with the percent being 35.8% among men and 64.2% among women (p < 0.05). Among the participants reporting drinking and driving, 4,691 indicated to do so "sometimes" (33.7% of men vs. 25.7% of women, p < 0.05, table 1), and the remaining 3,179 reported drinking and driving "almost never".

**Table 1:** Prevalence (%) of drinking and driving in the SUN cohort

	Men (N = 6,588)		Women (N = 9,613)	
	Sometimes (N = 2,220)	Sometimes + almost never (N = 4,229)	Sometimes (N = 2,471)	Sometimes + almost never (N = 3,441)
Overall	33.7 ^c^	64.2 ^c^	25.7	35.8
Age group				
<25	21.5^c^	31.6 ^c^	13.0	16.7
25–<30	31.7^b^	52.7 ^c^	26.8	35.4
30–<35	37.7^c^	67.5 ^c^	30.2	42.0
35–<40	40.3^c^	71.0 ^c^	32.2	45.4
40–<45	35.6^c^	69.5 ^c^	32.8	44.8
45–<50	33.7^b^	69.1 ^c^	26.8	38.0
>= 50	31.8^c^	67.3 ^c^	19.1	31.5
Marital status				
Single	31.3	54.6	23.8	33.8
Married	35.2	69.0	27.6	37.1
Widowed	31.0	65.5	17.3	28.2
Divorced/others	31.4	74.6	33.1	51.8
*p (chi square)*	*0.01*	*<0.001*	*<0.001*	*<0.001*
Health-related profession				
Medical Doctor	34.6	67.6	29.6	42.2
Nurse	40.7	75.4	28.1	38.8
Not health-related	33.2	62.8	23.9	33.1
*p (chi square)*	*0.06*	*<0.001*	*<0.001*	*<0.001*
Smoking (cig/d)				
Never smokers	32.9	57.2	22.3	30.0
<15	33.8	67.4	26.3	37.1
>= 15	32.6	66.0	29.4	42.8
Ex-smokers (>1 yr)	35.0	72.0	31.6	45.1
*p (chi square)*	*0.38*	*<0.001*	*<0.001*	*<0.001*
Body mass index (kg/m^2^)				
<25	32.9	59.1	26.1	36.1
>= 25	34.4	68.7	23.6	33.9
*p (chi square)*	*0.17*	*<0.001*	*0.05*	*0.09*
Average km driven/year				
<1,000	14.2	25.0	17.2	22.0
1,000–10,000	32.1	53.0	23.0	31.5
10,001–20,000	35.1	67.6	29.8	41.7
20,001–50,000	35.7	72.1	31.9	46.7
>50,000	34.8	70.2	30.4	45.3
*p for trend*	*<0.001*	*<0.001*	*<0.001*	*<0.001*
Use of seat belts				
Always	34.0	63.5	25.6	34.8
Not always	33.9	71.3	28.1	43.5
Never/Almost never	29.0	72.6	27.0	44.6
*p for trend*	*0.44*	*<0.001*	*0.09*	*<0.001*
Mean alcohol intake (g/d) according to FFQ				
0	17.6	25.7	11.9	15.4
<= 10	37.5	62.9	30.2	41.0
10–20	37.7	76.6	38.8	59.4
>20	28.4	80.9	29.4	62.1
*p for trend*	*0.06*	*<0.001*	*<0.001*	*<0.001*
Drinking pattern (days/wk)				
None or almost never	25.8	34.5	17.9	22.7
1–5	38.5	70.5	33.6	46.9
6–7	31.5	79.4	31.1	56.5
*p for trend*	*0.001*	*<0.001*	*<0.001*	*<0.001*
Binge Drinking (drinks/session)				
<= 5	32.0	58.5	23.8	32.5
>5 (some occasions)	36.3	72.8	32.3	47.3
*p (chi square)*	*0.001*	*<0.001*	*<0.001*	*<0.001*
History of traffic injury				
No	33.9	64.1	25.2	35.3
Yes	31.5	65.8	36.4	47.1
*p (chi square)*	*0.32*	*0.48*	*<0.001*	*<0.001*

FFQ: food frequency questionnaire

Fisher 2-tails test: ^a^p < 0.001; ^b^p < 0.01; ^c^p < 0.001 for the comparison between men and women.

Table 1 presents the percent of drivers reporting "sometimes" or "sometimes or almost never" drinking and driving, by gender and a number of other personal characteristics. Across personal and lifestyle habits, percentages of women reporting driving after drinking were lower than those of men. Among men, the percent reporting "sometimes" drinking and driving ranged from a lower 14.2% for those who drove less than 1000 kilometres per year to a high 40.7% amongst those who were nurses. Adding those who reported "almost never" drinking and driving shifted upwards the percentages of men to a low 25% (again, for those driving the least) to a high 76.6% amongst those who reported drinking a mean alcohol intake between 10 and 20 g/d. These percents were significantly different according to marital status, profession type, smoking habits, body mass index, drinking habits, driving habits (including safety belt use) and prior history of traffic-related injury.

The results of multivariate regression analyses used to investigate the joint effects of all these variables on the probability of reporting drinking and driving "sometimes" are presented in Table 2. The statistically significant increases in the likelihood of drinking and driving include the amount of kilometres driven on average per year – with ORs ranging from 2.62 (95%CI 1.74–3.94) if > 50,000 km/y to 2.78 (95%CI 1.97–3.91) -if 10,000–20,000 km/y), and the influence of alcohol intake in other contexts. For example, compared to those not drinking at all, those drinking less or equal than 10 g/d presented an OR of 2.17 of drinking and driving (95%CI 1.75–2.69), while those reporting drinking more than 20 g/d had an OR of 1.35 (95%CI 1.03–1.77). Those reporting drinking 1–5 days per week and those drinking 6–7 days per week, also had significantly higher ORs of drinking and driving than those not drinking OR 1.43 (95%CI 1.23–1.67). Compared to those who drunk less than six drinks per session, those drinking six or more presented ORs of 1.14 (95%CI 1.02–1.29) for drinking and driving.

**Table 2:** Variables independently associated with self-reported drinking and driving In the SUN cohort. The answer "Yes, sometimes" was considered as the outcome

	Men (n = 6,588)		Women (n = 9,613)	
	n	Multivariate OR (95% CI)	n	Multivariate OR (95% CI)
Age group				
<25	405	1 (ref.)	1482	1 (ref.)
25–<30	868	1.45 (1.08–1.94)	2266	2.09 (1.72–2.52)
30–<35	889	1.93 (1.44–2.59)	1661	2.64 (2.16–3.23)
35–<40	851	2.26 (1.68–3.05)	1266	2.85 (2.31–3.52)
40–<45	753	1.79 (1.32–2.44)	1018	2.83 (2.26–3.54)
45–<50	806	1.75 (1.29–2.39)	927	2.18 (1.72–2.75)
>= 50	2090	1.64 (1.23–2.18)	1111	1.60 (1.26–2.02)
Health-related profession				
Medical Doctor	1421	1.03 (0.90–1.18)	1359	1.12 (0.97–1.29)
Nurse	199	1.32 (0.96–1.80)	2342	1.23 (1.05–1.43)
Not health-related	5042	1 (ref.)	6030	1 (ref.)
Average km driven/yr				
<1,000	296	1 (ref.)	1336	1 (ref.)
1,000–10,000	1431	2.65 (1.87–3.76)	3954	1.26 (1.97–1.49)
10,001–20,000	2359	2.78 (1.97–3.91)	2583	1.64 (1.38–1.95)
20,001–50,000	2257	2.71 (1.94–3.86)	1644	1.73 (1.43–2.07)
>50,000	319	2.62 (1.74–3.94)	214	1.84 (1.31–2.58)
Use of seat belts				
Always	5762	1 (ref.)	8447	1 (ref.)
Not always	776	1.01 (0.86–1.19)	1136	1.19 (1.03–1.38)
Never/Almost never	124	0.86 (0.58–1.28)	148	1.09 (0.74–1.60)
Mean alcohol intake (g/d)				
0	791	1 (ref.)	2777	1 (ref.)
<= 10	3436	2.17 (1.75–2.69)	5880	2.36 (2.04–2.72)
10–20	1333	1.99 (1.55–2.55)	802	2.75 (2.21–3.42)
<20	1102	1.35 (1.03–1.77)	272	1.84 (1.33–2.55)
Drinking pattern (days/wk)				
None or almost never	1583	1 (ref.)	4769	1 (ref.)
1–5	3370	1.43 (1.23–1.67)	4252	1.66 (1.48–1.86)
6–7	1709	1.19 (0.99–1.44)	710	1.40 (1.13–1.73)
Binge Drinking (drinks/session)				
<= 5	4009	1 (ref.)	7594	1 (ref.)
>5 (some occasions)	2653	1.14 (1.02–1.29)	2137	1.20 (1.06–1.35)
History of traffic injury				
No	6221	1 (ref.)	9313	1 (ref.)
Yes	441	0.91 (0.73–1.12)	418	1.65 (1.33–2.05)
Smoking (cig/d)				
Never smokers	3043	1 (ref.)	5065	1 (ref.)
<15	681	1.03 (0.86–1.24)	1618	1.01 (0.88–1.16)
>= 15	623	1.00 (0.82–1.21)	698	1.15 (0.95–1.38)
Ex-smokers (>1 yr)	2315	1.10 (0.97–1.25)	2350	1.24 (1.10–1.40)

Among women, these results were also apparent (albeit with smaller variations in the specific OR values). However, among women other covariates showed an independent significant increased likelihood of reporting drinking and driving. Being a nurse was associated with an OR 1.23 (95%CI 1.05–1.43) as compared with having a non health-related profession. Not always wearing safety belts had an OR of 1.19 (95%CI 1.03–1.38) as compared to always using it. Presenting a history of traffic injury had an OR of 1.65 (95%CI 1.33–2.05) as compared to not having it. Being an ex-smoker (for at least one year), had an OR of 1.24 (95%CI 1.10–1.40) as compared to never smoking.

We repeated the same multivariate models, this time including in the outcome those reporting drinking and driving "almost never", only to find very similar results to those described above (data not shown). Last, we repeated these models excluding cohort participants who were not drivers. Table 3 summarizes the results. Again, findings are very similar to the ones already described, except that now all covariates reached statistical significance among both men and women and that ORs values had slightly higher magnitudes with many of them reaching values of 3.0 and 4.0. For example, among men drinking 6–7 days a week, the OR of drinking and driving increased to 4.48 (95%CI 3.64–5.51); for those between 35 and <40 years old the OR increased to 4.13 (95%CI 3.00–5.70); and for those driving between 20000 and 50000 km/year the OR was 4.06 (95%CI 2.91–5.65). Surprisingly, being a health professional (whether physician or nurse), as opposed to holding any other type of university degree, increased the OR to 1.22 if male physicians (95%CI 1.05–1.43), 2.05 if male nurse (95%CI 1.38–3.05), 1.2 if female physician (95%CI 1.04–1.38), and 1.27 if female nurse (95%CI 1.09–1.48). Compared to regular safety belt users, those not consistently using it presented ORs of drinking and driving of 1.61 (95%CI 1.39–1.86) or 1.84 (95%CI 1.50–2.25) depending on whether they were women or men, respectively.

**Table 3:** Variables independently associated with self-reported drinking and driving in the SUN cohort. Non-drivers were excluded. Either the answer "Yes, sometimes" or "Almost never" were considered as outcomes.

	Men (n = 6,365)		Women (n = 8,602)	
	n	Multivariate OR (95% CI)	N	Multivariate OR (95% CI)
Age group				
<25	318	1 (ref.)	1087	1 (ref.)
25–<30	817	1.57 (1.16–2.12)	2043	2,05 (1,71–2,47)
30–<35	860	3.30 (2.42–4.52)	1562	2,97 (2,44–3,61)
35–<40	837	4.13 (3.00–5.70)	1189	3,33 (2,70–4,10)
40–<45	735	3.24 (2.33–4.51)	945	3,03 (2,43–3,79)
45–<50	778	3.36 (2.41–4.68)	841	2,31 (1,84–2,90)
>= 50	2020	3.46 (2.56–4.69)	935	2,14 (1,70–2,68)
Health-related profession				
Medical Doctor	1388	1.22 (1.05–1.43)	1281	1,20 (1,04–1,38)
Nurse	196	2.05 (1.38–3.05)	2165	1,27 (1,09–1,48)
Not health-related	4781	1 (ref.)	5156	1 (ref.)
Average km driven/yr				
<1,000	223	1 (ref.)	1020	1 (ref.)
1,000–10,000	1300	2.64 (1.89–3.70)	3406	1,28 (1,09–1,51)
10,001–20,000	2304	3.58 (2.58–4.98)	2408	1,72 (1,45–2,04)
20,001–50,000	2224	4.06 (2.91–5.65)	1579	2,00 (1,67–2,40)
>50,000	314	3.71 (2.45–5.64)	189	2,54 (1,80–3,58)
Use of seat belts				
Always	5508	1 (ref.)	7473	1 (ref.)
Not always	735	1.84 (1.50–2.25)	999	1,61 (1,39–1,86)
Never/Almost never	122	1.67 (1.05–2.65)	130	1,79 (1,22–2,63)
Mean alcohol intake (g/d)				
0	735	1 (ref.)	2372	1 (ref.)
<= 10	3274	2.56 (2.09–3.13)	5242	2,57 (2,25–2,94)
10–20	1287	3.11 (2.42–4.00)	740	3,64 (2,93–4,52)
>20	1069	3.07 (2.32–4.06)	248	3,74 (2,70–5,19)
Drinking pattern (days/wk)				
None or almost never	1503	1 (ref.)	4181	1 (ref.)
1–5	3207	3.42 (2.91–4.00)	3772	2,12 (1,89–2,37)
6–7	1655	4.48 (3.64–5.51)	649	2,49 (2,02–3,08)
Binge Drinking (drinks/session)				
<= 5	3839	1 (ref.)	6714	1 (ref.)
>5 (some occasions)	2526	1.86 (1.62–2.14)	1888	1,47 (1,30–1,66)
History of traffic injury				
No	5939	1 (ref.)	8223	1 (ref.)
Yes	426	0.88 (0.69–1.11)	379	1,60 (1,28–2,02)
Smoking (cig/d)				
Never smokers	2889	1 (ref.)	4394	1 (ref.)
<15	642	1.19 (0.97–1.47)	1403	1,05 (0,92–1,20)
>+15	582	1.23 (0.99–1.54)	616	1,25 (1,04–1,51)
Ex-smokers (>1 yr)	2252	1.32 (1.14–1.53)	2189	1,27 (1,13–1,43)

**Figure 1:**
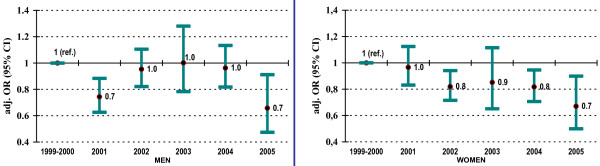
**Temporal trends in drinking and driving in the SUN cohort (non-drivers were excluded)**. Odds ratio estimates were computed after adjusting for categories of age, profession, average kilometres driven per year, use of safety belt, mean alcohol intake, drinking pattern, binge drinking, history of traffic injury and smoking status.

Adding cohort enrolment year to these multivariate regression models to investigate whether the prevalence of drinking and driving among subjects enrolling in recent years produced the results summarized in Figure 1. Among women, we observed a statistically significant trend with participants joining the cohort in more recent years presenting lower prevalence of drinking and driving than those enrolled in earlier years (e.g., OR: 0.70, 95% CI: 0.50–0.85 among women enrolling during 2005). However, this effect was not apparent in men.

## Discussion

Our findings reveal an extremely high prevalence of self-reported drinking and driving among a high education segment the Spanish participants in the SUN cohort study. Only slightly more than half our participants (53%) reported "never" drinking and driving, and up to 30% of the participants acknowledged to drink and drive "sometimes". Although our cohort is not expected to be representative of the Spanish educated population, this high prevalence seems to be also spreading to the general Spanish population if one is to use the 2002 SARTRE-3 unpublished findings or studies from other countries [4,8,12,14-19].

In particular, and even though our sample is not comparable on educational level to those in some US studies [4,12,14] our findings confirm the impact of age and gender on the prevalence of this practice. Our findings also confirm the impact of educational level seen in other studies [15,18]. Probably, the effect of age on increasing the prevalence is what justifies that our findings differ from those in studies with younger populations, such as those derived from university students [16,17], including an European study [19], although it may also be that the wording of the questions used in those studies (e.g., "within the past 30 days how many times [have you] driven when [you] have had perhaps too much to drink") elicits more conservative answers. It also confirms the trends observed in 4 of 7 countries around the world [8].

More importantly, the strengths of cohort studies reside on the investigation of relationships between variables and the follow-up data that they can provide. Our study found that drinking and driving was also related to other unsafe practices. Not surprisingly, binge drinking and daily drinking were also more common amongst those who reported drinking and driving, as seen in US studies [23], where 80% of drink and drive episodes are reported by those who also binge drink [11], or where drunk drivers are also more than twice likely to report daily drinking [16]. In our study, drivers who have drunk were also less likely to use their safety belts, a finding also reported in [4,16,19]. Although we did not investigate this issue, it is noteworthy to mention here that drivers who had been drinking were less likely to restrain their children [24]. Rarely investigated in other analytical studies, our findings also highlight the higher prevalence of drinking and driving amongst those who are more exposed to traffic since they drive the most.

The elevated prevalence rates of drinking and driving of the educated population included in our study may seem in conflict with other studies, where high income and education levels have proved to have a protective effect on alcohol-related fatal motor vehicle crashes [25]. However, those studies have not investigated whether this effect is so because more educated and/or wealthier individuals drink less or because of other issues such as better health status pre accident, better vehicles, driving in better roads (e.g., highways) or receiving better health care.

Never reported before is our finding that health professionals (physicians or nurses) are more likely to report drinking and driving than other university graduates even after controlling for possible confounders. The role of these health professionals in educating the population regarding the health consequences of drinking and of drinking and driving has been long advocated for [9,26,27]. Yet, their capability to do so may be impaired due to their own lifestyles. Health professionals act as models for their patients and this can be seen, for example, in smoking cessation programmes. Several reports suggest that when an appropriately-trained physician provides counselling and guidance, smoking cessation success rates can be increased [28-30]. However, it has been found that anywhere from 30–63% of smokers had not received cessation counselling from their physicians within the last year [31]. Physicians' own smoking patterns and quitting behaviours are important because physicians serve as models for their patients and play a key role in the reinforcement of smoke-free health facilities [32]. It can be difficult to be a patient's model and have more self-confidence in the success of patients' smoking counselling if the frequency of smoking among doctors is high, as it has been shown in Italy [33]. In fact, as frequency of smoking decreases in doctors they try to convince more people to stop smoking. In a comparison between Finnish (smoking prevalence of 6.7% in males) and Estonian physicians (smoking prevalence 18.6% in males), Finnish doctors with a much lower frequency of smoking, are more likely to believe that it is their responsibility to help patients stop smoking [32]. Some similar attitudes have been observed regarding health education provided by nurses. Nurses who smoke are less motivated to provide cessation support for patients, have less positive attitudes to the value of smoking cessation, are less likely to have received smoking cessation training and are less likely to want further training [34]. How does this tobacco-based evidence translate into the motor vehicle safety world remains a subject for further investigation, but our current study suggests the possibility that traffic safety practices may not be well addressed by health professionals.

Also never reported for Europe is our finding that the prevalence of self-reported drinking and driving seems to be diminishing in more recent years, at least among women. Whether this is a true trend or simply a reflection of an increase in socially desirable answers to the question related to drinking and driving remains to be investigated. It could also be the case that subjects enrolling in the cohort in more recent years are substantially different from the earlier participants. However, since our sampling framework has not changed and neither has changed our invitation protocol, we do not believe this to be an explanation for this finding. Analyses of US data from 1983 to 2003 suggest that there has been a decrease in the overall percent of drinking and driving among motor vehicle drivers and motorcyclist [35]. This reduction is particularly true among younger individuals, which has turned into a shift in the peak age with the highest rates of drinking and driving from the 20–24 years old to those between 40–44 years old. This evidence about the reduction in the prevalence of drinking and driving over time is mentioned in several other publications as possible reasons to explain the reductions of motor vehicle alcohol-related deaths in the US up to 2001 [36]. However, other studies are pointing out to increases in alcohol impaired driving both in the US [11] and in European countries [8], such as Finland [37]. As our cohort continues to grow due to its open enrolment nature, we will continue to monitor these trends.

As with other public health problems, an array of interventions to reduce the prevalence of alcohol amongst road users has been set in practice around the world, including legislation prohibiting driving at or over certain levels of alcohol in blood, or the recommendation that advice on this matter is provided in the context of routine general medical visits [26]. Most of these measures are in place in Spain. However, in light of these findings and others in the literature, it seems as if much more needs to be done.

## Conclusion

Our findings are amongst the first on the high prevalence of drinking and driving among Spanish. Particularly worrisome is the fact that health professionals reported this habit even at higher rates. Multidisciplinary interventions (e.g., legal, educational, economic) are needed to reduce this serious health risk.

## Competing interests

The author(s) declare that they have no competing interests.

## Authors' contributions

MSG conducted some of the analysis, drafted the first manuscript and incorporated all comments and suggestions. SP collaborated in the literature review and the interpretation of results, FG and JI made substantial comments to the earlier analyses findings and manuscript drafts, and so did MMG, who also was involved in the statistical analysis.

All authors read and approved the final manuscript.

All authors confirm that the content has not been published elsewhere and does not overlap or duplicate their published work.

## Pre-publication history

The pre-publication history for this paper can be accessed here:


